# Cohort profile: the British Columbia COVID-19 Cohort (BCC19C)—a dynamic, linked population-based cohort

**DOI:** 10.3389/fpubh.2024.1248905

**Published:** 2024-02-21

**Authors:** James Wilton, Jalud Abdulmenan, Mei Chong, Ana Becerra, Mehazabeen Najmul Hussain, Sean P. Harrigan, Héctor Alexander Velásquez García, Zaeema Naveed, Hind Sbihi, Kate Smolina, Marsha Taylor, Binay Adhikari, Moe Zandy, Solmaz Setayeshgar, Julia Li, Younathan Abdia, Mawuena Binka, Drona Rasali, Caren Rose, Michael Coss, Alexandra Flatt, Seyed Ali Mussavi Rizi, Naveed Zafar Janjua

**Affiliations:** ^1^BC Center for Disease Control, Provincial Health Services Authority, Vancouver, BC, Canada; ^2^Data Analytics, Reporting, and Evaluation, Provincial Health Services Authority, Vancouver, BC, Canada; ^3^Trauma Services British Columbia, Provincial Health Services Authority, Vancouver, BC, Canada; ^4^Vancouver Coastal Health, Vancouver, BC, Canada; ^5^School of Population and Public Health, University of British Columbia, Vancouver, BC, Canada; ^6^Faculty of Pharmaceutical Sciences, University of British Columbia, Vancouver, BC, Canada; ^7^Provincial Health Services Authority, Vancouver, BC, Canada; ^8^Centre for Health Evaluation and Outcome Sciences, St. Paul's Hospital, Vancouver, BC, Canada

**Keywords:** COVID-19, cohort profile, population-based data, administrative data, linked data

## Abstract

**Purpose:**

The British Columbia COVID-19 Cohort (BCC19C) was developed from an innovative, dynamic surveillance platform and is accessed/analyzed through a cloud-based environment. The platform integrates recently developed provincial COVID-19 datasets (refreshed daily) with existing administrative holdings and provincial registries (refreshed weekly/monthly). The platform/cohort were established to inform the COVID-19 response in near “real-time” and to answer more in-depth epidemiologic questions.

**Participants:**

The surveillance platform facilitates the creation of large, up-to-date analytic cohorts of people accessing COVID-19 related services and their linked medical histories. The program of work focused on creating/analyzing these cohorts is referred to as the BCC19C. The administrative/registry datasets integrated within the platform are not specific to COVID-19 and allow for selection of “control” individuals who have not accessed COVID-19 services.

**Findings to date:**

The platform has vastly broadened the range of COVID-19 analyses possible, and outputs from BCC19C analyses have been used to create dashboards, support routine reporting and contribute to the peer-reviewed literature. Published manuscripts (total of 15 as of July, 2023) have appeared in high-profile publications, generated significant media attention and informed policy and programming. In this paper, we conducted an analysis to identify sociodemographic and health characteristics associated with receiving SARS-CoV-2 laboratory testing, testing positive, and being fully vaccinated. Other published analyses have compared the relative clinical severity of different variants of concern; quantified the high “real-world” effectiveness of vaccines in addition to the higher risk of myocarditis among younger males following a 2nd dose of an mRNA vaccine; developed and validated an algorithm for identifying long-COVID patients in administrative data; identified a higher rate of diabetes and healthcare utilization among people with long-COVID; and measured the impact of the pandemic on mental health, among other analyses.

**Future plans:**

While the global COVID-19 health emergency has ended, our program of work remains robust. We plan to integrate additional datasets into the surveillance platform to further improve and expand covariate measurement and scope of analyses. Our analyses continue to focus on retrospective studies of various aspects of the COVID-19 pandemic, as well as prospective assessment of post-acute COVID-19 conditions and other impacts of the pandemic.

## Introduction

BC is the Western-most province of Canada and has a population of approximately 5.2 million people (as of 2022). BC recorded its first COVID-19 case on Jan 28, 2020 and by the end of 2022 had recorded approximately 390,000 laboratory confirmed infections, 31,000 hospitalizations and 4,800 deaths related to COVID-19 (1). Canada, and BC specifically, has had one of the highest COVID-19 vaccination rates in the world, with ~90% of adults receiving at least two doses within a year of vaccine rollout (2). As of the end of 2022, ~63% of adults had received a third dose and ~36% a fourth dose. Despite the active response to the pandemic, there has been and remains an ongoing need for innovative data platforms/environments to evaluate and inform the public health response in “real-time” and also support more in-depth epidemiologic questions about COVID-19 (3, 4).

The BC COVID-19 Data Library (BCCDL) surveillance platform was created at the beginning of the COVID-19 pandemic to improve access to linkable population-based datasets and support surveillance and public health planning (3). The BC COVID-19 Cohort (BCC19C) represents a program of work focused on developing analytic cohorts from the platform in order to meet these overall goals. The BCCDL and BCC19C are the result of a collaboration between provincial-level organizations, including the Health Sector Information, Analysis and Reporting (HSIAR) team at the BC Ministry of Health (MOH) and the Data Analytics, Reporting, and Evaluation (DARE) team and the British Columbia Centre for Disease Control (BCCDC) at the Provincial Health Services Authority (PHSA). The primary objectives of the BCCDL and BCC19C are:

° To support ongoing surveillance, reporting, and modeling of COVID-19 in BC;
° To describe and characterize the epidemiology of COVID-19 in BC;
° To inform acute care planning, public health interventions, and the implementation of health care services to address COVID-19 in BC.

## Methods and analysis

### Who is in the cohort?

**Figure 1:**
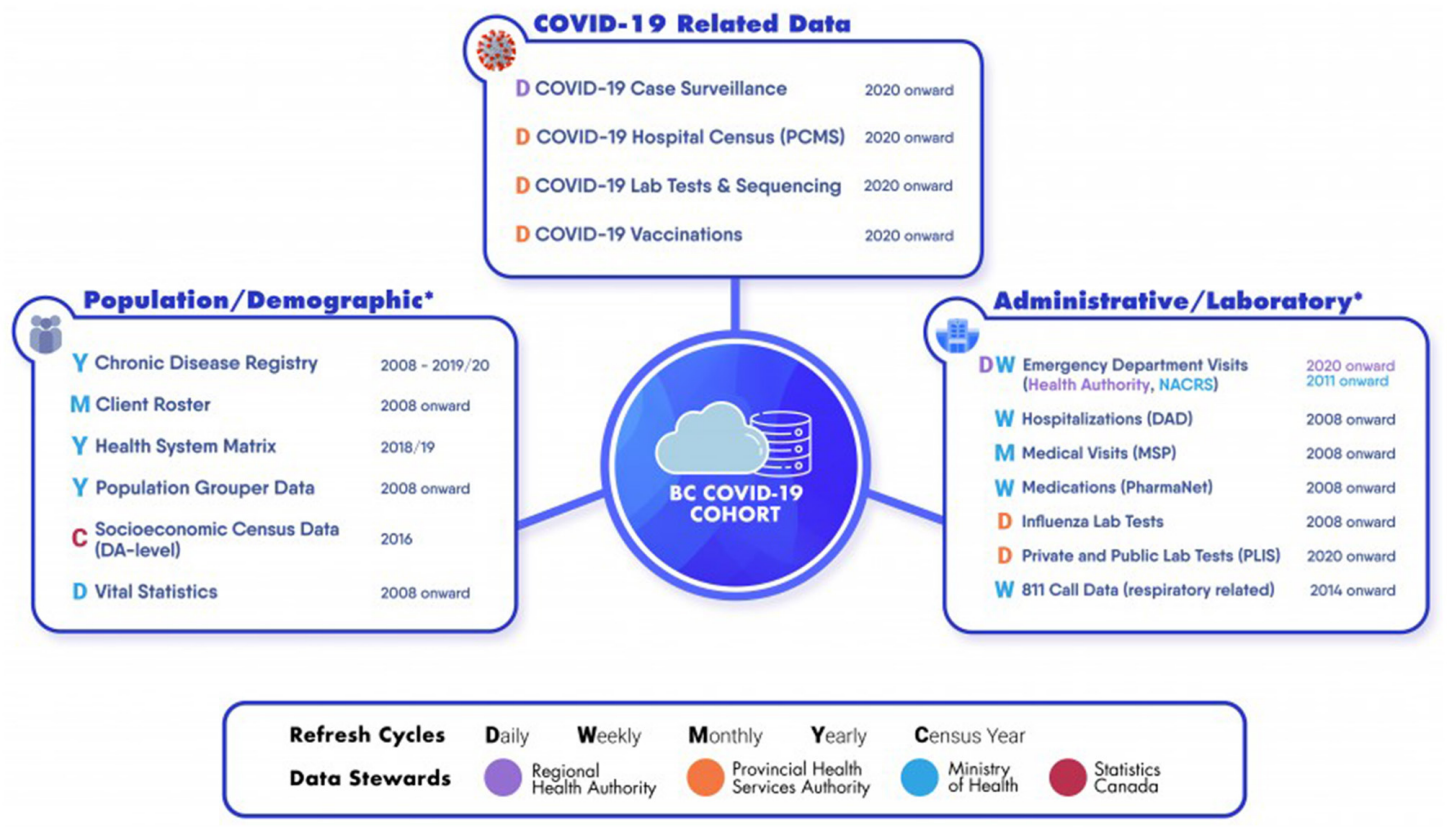
BC COVID-19 Cohort and its integrated datasets. The platform used to create the BC COVID-19 Cohort contains a range of datasets that differ by domain, Data Steward and refresh frequency. See Table 1 for more detailed descriptions of datasets.

The BCCDL is a cloud-based, dynamic surveillance platform that integrates recently developed COVID-19 datasets with already established administrative data holdings and other provincial registries (Figure 1 and Supplementary Table 1). Each dataset integrated within the platform has its own scope and most are population-based (i.e., cover all BC residents). While the COVID-19 datasets are by definition limited to people who have accessed COVID-19 services, the administrative datasets and provincial registries are not specific to COVID-19 and capture the population's overall use of health care services. The platform is dynamic and new individuals—as well as new records for existing individuals—are added with each dataset refresh. COVID-19 datasets are generally refreshed daily while administrative datasets and other registries are generally refreshed weekly/monthly (Table 1 and Figure 1).

**Table 1:** Description of linkable datasets.

**Data source**	**Description**	**Refresh frequency**	**Date range**	**Key measurements (outcomes, covariates, potential confounders)**
**COVID-19 datasets**				
Integrated lab dataset	COVID-19 laboratory tests conducted by private and public laboratories across BC. Includes RT-PCR diagnostic test results as well as results from single nucleotide polymorphism screening and whole genome sequencing.	Daily	January 2020–onward	SARS-CoV-2 case status, viral lineage/variant of concern
Case surveillance	Information on confirmed and epi-linked cases sent to BCCDC on a daily basis by the individual regional health authorities. These case data were compiled into a single line-list at the BCCDC.	Daily	January 2020–March 31 2022	SARS-CoV-2 case status, sociodemographics (sex, age, geography), severity of infection (hospitalization, ICU admission, death)
Vaccinations	Provincial Immunizations Registry covering all COVID-19 vaccinations administered in BC, in addition to some doses administrated out of province.	Daily	December 2020–onward	Vaccination status, vaccine type, vaccine dose
Provincial COVID-19 monitoring solution	Daily hospital census data from acute care facilities across the province.	Daily	January 2020–onward	Severity of infection (hospitalization, critical care/ICU admission), interventions/treatments (e.g., mechanical ventilation), length of stay
**Administrative health datasets, laboratory data, provincial registries**				
Client roster	Demographic and geographic information for individuals accessing the publicly funded universal health insurance program (Medical Services Plan).	Monthly	2008/09–onward	Sociodemographics (sex, age, geography)
Medical services plan	Records of all fee-for-service provider visits billed to the province's universal health insurance program. Includes outpatient visits to physician offices and some services provided in inpatient settings.	Daily/monthly^a^	2008/09–onward	Healthcare utilization, health conditions and co-morbidities.
Discharge abstract database	Captures discharges, transfers, and deaths occurring in acute care hospitals in BC.	Weekly	2008/9–onward	Severity of infection (hospitalization, ICU admission); length of stay; healthcare utilization, health conditions and co-morbidities; interventions/treatments (e.g., medical procedures/supports)
Emergency department visits	Emergency department visit data sent to the BCCDC by the individual regional health authorities. Incomplete provincial coverage.	Daily	March 2020–onward	Severity of infection; healthcare utilization, health conditions and co-morbidities
National ambulatory care reporting system	A national database designed to capture information on patient visits to hospital-based and community-based ambulatory care. Incomplete provincial coverage.	Weekly	2011/12–onward	Severity of infection; healthcare utilization, health conditions and co-morbidities
Provincial laboratory information solution	Comprehensive diagnostic laboratory test results from private and public laboratories across British Columbia (captures 95% of private/public laboratory tests in province).	Daily	January 2020–onward	Health conditions and co-morbidities
Respiratory data mart	Respiratory disease test results (e.g., influenza, RSV). Incomplete provincial coverage.	Daily	January 2008–onward	Health conditions and co-morbidities (respiratory only)
PharmaNet	An online, real-time system that captures all prescriptions for drugs and medical supplies dispensed from community pharmacies in the province.	Weekly	2008/9–onward	Health conditions and co-morbidities; interventions/treatments (e.g., nirmatrelvir/ritonavir)
Vital statistics	The Vital Statistics Agency registers all deaths that occur in British Columbia.	Daily	2008–onward	Severity of infection (death)
Chronic disease registry	Derived flags for chronic health condition for all individuals registered in the province's universal health insurance program (Medical Services Plan). Based on standard provincial algorithms that draw upon other administrative datasets with information on physician visits, hospitalizations and medication dispensations.	Annual	2019/2020	Health conditions and co-morbidities
Health system matrix	The BC Ministry of Health's approach to understanding the complexity and patterns of health care needs in a population. Summarizes information from multiple databases into a single dataset with derived, individual-level variables reflecting frequency/intensity of healthcare use.	Annual	2018/2019	Healthcare utilization, health conditions and co-morbidities
Population grouper methodology	Developed by the Canadian Institute of Health Information. Draws upon multiple data sources to build clinical, disease, and demographic profiles at the individual-level to better understand the population's healthcare needs.	Monthly to annual	2008/09–onward	Healthcare utilization, health conditions and co-morbidities
811 call data (respiratory and COVID-19 related calls)	A free-of-charge health information/advice phone line operated by HealthLink BC, which is part of the Ministry of Health. Patients can receive assessment of symptoms and, if necessary, information on testing and other next steps.	Bi-weekly	2014–onward	
Statistics Canada census of population	Geographic-level data from the Statistics Canada Census. Available at the level of the Dissemination Area (approx. 400–700 people).	5 years	2016	Socioeconomic status (e.g., household income, employment, education).

^a^There are two Medical Service Plan datasets: Unadjudicated (available on a more timely basis and is refreshed daily) and adjudicated (more lagged and is refreshed monthly). For more information on the Chronic Disease Registry, Health System Matrix, and Population Grouper Methodology datasets see http://www.bccdc.ca/resource-gallery/Documents/Chronic-Disease-Dashboard/BC%20CDR%20Methodology%20Overview%202018-19.pdf, https://www2.gov.bc.ca/assets/gov/health/forms/5511datadictionary.pdf and https://www.cihi.ca/sites/default/files/document/cihi-population-grouping-methodology-v1_3-overview-outputs-manual-en.pdf, respectively.

The BCC19C represents the analytic cohorts developed from the platform. These cohorts primarily include individuals accessing COVID-19 related services (e.g., testing or vaccination) and their linked medical histories. As of the end of 2022, the number of unique individuals who had received at least one RT-PCR diagnostic test or vaccine dose for COVID-19 in BC was 2.25 million and 4.65 million, respectively. These individuals can be linked to administrative datasets and provincial registries that contain information on sociodemographics, healthcare use, comorbidities, and other covariates and outcomes (Table 1). In addition, the administrative/registry datasets integrated within the platform allow for selection of “control” individuals who have not accessed COVID-19 related services.

All datasets are de-identified but individuals can be linked across the datasets using a unique, randomly assigned patient master key (PMK). The de-identification process involves the removal of all direct identifiers, including name, address, Personal Health Number (PHN), and full date of birth (imputed to first of month). In brief, the patient matching algorithm/service used to assign the PMK is based on a series deterministic and probabilistic fuzzy matching to link patient records within and across different data sources and assign a PMK (5). The first step of algorithm is deterministic matching using PHN (a unique lifetime identifier assigned to individuals enrolled in BC's universal public healthcare plan) and then non-deterministic fuzzy matches (based on non-PHN variables) within PHN sets. A series of deterministic and non-deterministic matches are then applied for patients without a PHN based on sex, full date of birth, and name (first, middle and last). The patient matching algorithm has been developed and refined over the past 12 years and is regularly monitored and adjusted for accuracy. Each step was validated with manual review of random samples of data and match rates based on this review were between 99 and 100% (depending on dataset). The patient matching algorithm requires review, validation, and recalibration after introduction of new datasets. Limitations include the potential for errors in-between validation cycles, the large number of records without a PHN, limited availability of non-PHN variables (e.g., address is not universally available or standardized) and poor data quality of these non-PHN variables. The patient matching service is run daily on more than 65 million records each day in ~3 h.

### What data sources are available and what can be measured?

Each dataset integrated within the platform has its own date range, refresh schedule, and available data elements (Figure 1 and Table 1).

The COVID-19 datasets contain information on individuals tested, diagnosed, hospitalized and vaccinated for COVID-19. The SARS-CoV-2 laboratory testing data includes positive and negative RT-PCR diagnostic tests as well as viral lineage information obtained from single nucleotide polymorphism (SNP) screening and/or whole genome sequencing. Viral mutation-level genomic data are also available (publicly available in GISAID under the submitter: BCCDC PHL). Case surveillance data includes information obtained by the regional health authorities during case follow-up (e.g., sociodemographics, exposure history, hospital admission, death). The Provincial COVID-19 Monitoring Solution (PCMS) is a daily census of acute care hospitals across BC and contains information on admission and discharge dates, unit of care (e.g., intensive care unit), supports used (e.g., mechanical ventilation) and discharge status (e.g., alive, dead). The COVID-19 vaccination dataset captures information on date of administration, trade name, dose and dose number for all vaccines administered in BC, as well as doses administered to BC residents outside of BC as reported by the vaccinated individual.

Several provincial-level administrative health datasets and registries are available for linkage to the COVID-19 datasets. The Client Roster includes all individuals accessing medical care through publicly funded health insurance and captures individual-level demographic data (e.g., imputed birth dates, geography). Geographic-level socioeconomic status (SES) and social determinants of health (SDOH) data are available from the Statistic Canada Census of Population that is conducted every 5 years (available at level of Dissemination Area—a uniform population size of between 400–700 people). Information on inpatient and outpatient medical visits (e.g., physician office, hospital, emergency department), deaths, medication dispensations, and laboratory diagnostic tests can be used to determine comorbidities and monitor healthcare use. These datasets can also be used to complement COVID-specific datasets in identifying severe outcomes related to SARS-CoV-2 infection (e.g., hospitalizations, deaths). Also available are composite datasets (Chronic Disease Registry, Health System Matrix, Population Grouper Methodology) that use standardized methods to derive information from administrative data holdings. The Chronic Disease Registry contains individual-level metrics on lifetime history of co-morbidities, while the Health System Matrix and Population Grouper Methodology classify individuals into categories based on healthcare utilization and/or comorbidity history.

### How are data accessed, stored and governed?

The platform data are primarily stored and securely housed in a cloud-based, Microsoft Azure SQL Database environment—a fully managed database service in Azure. Datasets are received from Data Stewards/source systems primarily in CSV format and initially stored in our data warehouse, cloud-based SQL servers (Microsoft Azure—Infrastructure as a Service, IAAS). The datasets proceed through automated extract, transform and load (ETL) jobs in the data warehouse and are subsequently pushed to a separate cloud-based SQL Database Environment (Microsoft Azure—Software as a Service, SAAS) using Azure Data Factory pipelines. The ETL process for each dataset involves de-identification, exclusion of data not approved for broad use, standardization of variable names, and assignment of data types. Data housed in the SAAS servers can then be accessed by authorized users through pooled virtual machines which are pre-installed with various analytic and visualization software (e.g., SAS, R, SQL Server Management Studio, Power BI). This environment facilitates linkage between datasets by housing them all in a single location. The database also provides a read/write schema where users can store tables created during their analyses.

There are several challenges to our approach to data storage and access. Due to large size of datasets and network/resource limitations, the initial ETL pipeline cannot all be run in the final cloud-based Azure Database Environment (but must first be run in the data warehouse servers) and must also be scheduled separately for each dataset (as opposed to occurring simultaneously for all datasets). The ETL processes are run overnight and datasets cannot be queried by users while in progress. Further, the large datasets can be resource intensive to manipulate/query by users when conducting epidemiological analyses, and therefore cost-benefit decisions are required to balance the impact of resource limitation issues (e.g., crashing/slowing of virtual machines) with the cost of provisioning additional memory. Finally, the ETL process involves minimal data cleaning and therefore additional educational and technical support must be provided to users to ensure adequate/accurate manipulation and use of available data.

The platform went through a Privacy Impact Assessment and is governed under the terms of an Information Sharing Agreement (ISA) with the BC MOH and initially by a PHSA COVID-19 Analytics Network (now called the Viral Respiratory Infection Analytics Network). The Network was established to review the proposed uses of the platform, ensure projects align with the needs of various PHSA stakeholders, and act as a community of practice for sharing findings and enhancing collaboration among partners The Network has also been an important forum for awareness of other provincial, national and international COVID-19 efforts. The platform's centralized approach to data access ensures all agreements (e.g., ISAs) with Data Stewards, governance, and security infrastructure are already in place for data requestors. This streamlined model reduces the responsibility on individual requestors to separately coordinate these tasks with Data Stewards. Authorized users have access to de-identified data based on roles and projects and must agree to Terms of Use and complete pre-requisite privacy, confidentiality, and security courses. Public dissemination of each data product (e.g., report, manuscript) requires Data Steward pre-approval and additional assessment to ensure alignment with PHSA and Data Steward risk of re-identification policies (e.g., suppression of small cell sizes).

## Findings to date

There are several areas of focus for BCC19C analyses. A summary of completed and ongoing analyses by these areas are found below and a list of published analyses can be found here: https://a4ph.med.ubc.ca/publications-and-presentations/publications/.

### Cohort profile

To create a cohort profile, we conducted several comparisons (tested vs. never tested, tested positive vs. tested negative, fully vaccinated with 2 or more doses vs. not) to characterize individuals in the BCC19C up to November 30th, 2021 (pre-Omicron; Table 2). In addition to highlighting the breadth of data available, these comparisons demonstrate the importance of the administrative datasets and provincial registries for assigning sociodemographics; measuring history of health conditions and healthcare use; and identifying “control” groups for comparison to people who have accessed COVID-19 services (e.g., people who have not received laboratory testing for COVID-19).

**Table 2:** Characteristics of individuals in the BC COVID-19 Cohort (tested vs. never tested, tested positive vs. tested negative, fully vaccinated vs. not) during the pre-Omicron period (to Nov 30th, 2021).

	**Ever tested for COVID-19**			**Ever tested positive for COVID-19 (among tested)**			**Fully vaccinated for COVID-19 (two or more doses)**		
	**No (*N =* 2,801,651)**	**Yes (*N =* 1,673,214)**	**aOR (95%CI)**	**No (*N =* 1,500,856)**	**Yes (*N =* 172,358)**	**aOR (95%CI)**	**No (*N =* 754,227)**	**Yes (*N =* 3,295,485)**	**aOR (95%CI)**
	**Column %**	**Column %**		**Column %**	**Column %**		**Column %**	**Column %**	
**Sex**									
Female	49.7	53.7	1.2 (1.1–1.2)	54.1	50.1	0.9 (0.8–0.9)	47.1	52.5	1.1 (1.1–1.1)
Male	50.3	46.3	Ref	45.9	49.9	Ref	52.9	47.5	Ref
**Age (years)**									
0–4	2.1	2.3	3 (3–3.1)	2.4	1.4	1 (0.9–1)	-	-	-
5–11	6.8	8.4	3.5 (3.5–3.5)	8.3	8.4	1.6 (1.5–1.6)	-	-	-
12–17	6.5	6.6	2.8 (2.8–2.8)	6.6	6.6	1.5 (1.5–1.5)	8.9	6.8	0.7 (0.7–0.7)
18–29	12.9	18.3	3.7 (3.7–3.7)	17.9	22.0	1.8 (1.8–1.9)	20.1	15.7	0.7 (0.7–0.7)
30–39	12.3	18.2	3.7 (3.6–3.7)	18.1	18.9	1.6 (1.6–1.7)	19.6	15.2	0.7 (0.6–0.7)
40–49	11.8	13.6	2.8 (2.7–2.8)	13.4	15.1	1.8 (1.7–1.8)	14.7	13.5	0.7 (0.7–0.8)
50–59	13.7	11.9	2 (2–2)	11.8	12.1	1.6 (1.6–1.7)	14.2	14.4	0.8 (0.8–0.8)
60–69	15.2	9.9	1.4 (1.4–1.4)	10.1	8.2	1.3 (1.3–1.3)	11.4	15.3	1 (0.9–1)
70+	18.9	10.9	Ref	11.3	7.1	Ref	11.1	19.1	Ref
**Material deprivation (quintiles)**									
1 (least)	17.7	20.0	Ref	20.6	14.8	Ref	15.6	19.5	Ref
2	22.4	23.2	0.9 (0.9–0.9)	23.6	19.4	1 (1–1.1)	20.3	23.0	0.9 (0.9–1)
3	21.2	20.1	0.8 (0.8–0.8)	20.2	18.6	1.1 (1.1–1.2)	21.6	20.5	0.8 (0.8–0.8)
4	20.2	18.3	0.8 (0.8–0.8)	18.0	20.8	1.4 (1.4–1.5)	21.7	18.9	0.7 (0.7–0.7)
5 (most)	14.2	13.7	0.8 (0.8–0.8)	12.8	21.8	2 (2–2.1)	15.7	13.6	0.7 (0.7–0.7)
Missing	4.4	4.8	1 (1–1)	4.8	4.8	1.4 (1.4–1.5)	5.2	4.5	0.7 (0.7–0.7)
**Social deprivation (quintiles)**									
1 (least)	18.2	19.6	-	19.1	24.0	-	16.4	19.0	-
2	20.7	20.6	-	20.6	21.1	-	19.2	20.8	-
3	18.9	18.0	-	18.2	16.6	-	18.1	18.6	-
4	18.5	17.8	-	18.0	16.0	-	18.9	18.1	-
5 (most)	19.3	19.2	-	19.4	17.6	-	22.3	19.0	-
Missing	4.4	4.8	-	4.8	4.8	-	5.2	4.5	-
**Regional health authority**									
Fraser	32.9	43.1	Ref	42.2	50.7	Ref	34.1	36.7	Ref
Interior	17.7	15.3	0.7 (0.7–0.7)	15.3	15.6	0.9 (0.9–0.9)	20.8	16.1	0.7 (0.7–0.7)
Northern	6.8	4.9	0.6 (0.5–0.6)	4.5	8.7	1.6 (1.6–1.7)	9.1	5.2	0.5 (0.5–0.5)
Vancouver coastal	23.0	23.0	0.8 (0.7–0.8)	23.5	18.7	0.7 (0.7–0.7)	20.9	23.8	1 (1–1)
Island	19.6	13.7	0.5 (0.5–0.5)	14.6	6.4	0.4 (0.4–0.4)	15.1	18.2	1 (1–1)
**Population segment (HSM)**									
Non-user	15.6	10.2	0.5 (0.5–0.5)	10.0	11.4	1 (1–1)	23.8	11.1	0.4 (0.4–0.4)
Healthy (low user)	33.7	40.2	Ref	39.9	42.4	Ref	31.2	34.3	Ref
Adult major age 18+	3.0	3.3	1.2 (1.2–1.2)	3.3	3.2	1 (0.9–1)	2.7	3.6	1.2 (1.2–1.2)
Child and youth major < 18 years	0.6	0.9	1.1 (1.1–1.1)	0.9	0.8	1 (0.9–1)	0.3	0.3	0.9 (0.9–1)
Low chronic conditions	25.2	25.9	1.2 (1.2–1.2)	26.0	25.6	1 (0.9–1)	21.9	28.6	1.1 (1.1–1.1)
Medium chronic conditions	10.1	7.7	1.4 (1.4–1.4)	7.9	6.0	0.9 (0.9–0.9)	5.8	11.2	1.5 (1.4–1.5)
Severe mental health and substance use	1.6	2.4	1.5 (1.5–1.5)	2.4	2.7	1 (1–1)	3.5	1.8	0.5 (0.5–0.5)
Maternity and healthy newborns	1.6	2.9	1.2 (1.2–1.2)	2.9	2.6	0.9 (0.9–1)	1.8	1.5	0.8 (0.8–0.8)
High chronic conditions	4.6	4.3	1.9 (1.9–1.9)	4.4	3.4	1 (0.9–1)	2.8	5.5	1.4 (1.4–1.5)
Cancer	1.5	1.5	1.5 (1.5–1.5)	1.6	1.2	0.8 (0.8–0.8)	1.0	1.7	1.4 (1.4–1.4)
Frail in long-term care	0.2	0.5	6.7 (6.5–7)	0.5	0.6	1.5 (1.4–1.6)	0.1	0.4	3.3 (3–3.5)
End of life	0.1	0.1	2.2 (2–2.3)	0.1	0.1	0.8 (0.7–1)	0.1	0.1	1.2 (1.1–1.4)
Missing	2.3	0.1	0 (0–0)	0.1	0.1	0.6 (0.5–0.7)	5.2	0.0	0 (0–0)
**Number of physical health conditions (CDR)**									
0	59.7	62.4	-	62.2	64.5	-	69.6	54.7	-
1–2	30.7	28.7	-	28.8	28.4	-	25.4	33.8	-
3+	9.6	8.8	-	9.0	7.1	-	5.1	11.5	-
**Number of mental health conditions, including substance use (CDR)**									
0	68.6	63.1	-	62.8	66.3	-	65.5	62.6	-
1–2	28.0	32.0	-	32.4	28.6	-	27.9	33.5	-
3+	3.5	4.9	-	4.8	5.2	-	6.6	3.9	-

aOR, adjusted odds ratio; CI, confidence interval; CDR, chronic disease registry; HSM, health system matrix. Analysis includes testing, case and vaccination data to November 30th, 2021 (pre-Omicron period) and limited to individuals identified in client roster as being a BC resident in 2018/2019, 2019/2020, and 2020/2021 fiscal years. We excluded individuals who died on or prior to November 30th, 2021; received a non-mRNA vaccine or vaccine administered out of vaccine during study period; or had missing data on sex, age or geography. Latest HSM and CDR data were from the 2018/2019 and 2019/2020 fiscal year, respectively, leading to some misclassification. Odd ratios in table were derived from three separate multivariable logistic regression models assessing factors associated with “Yes” for the three different dependent variables (ever tested, diagnosed, fully vaccinated). All covariates in table were included in the multivariable models, with exception of social deprivation and number of health conditions—due to collinearity with other variables in model. Children < 12 years of age were excluded from vaccination model as vaccination had not yet been rolled out in this population. Physical health comorbidities included circulatory diseases, respiratory diseases, neurological disorders, musculoskeletal disorders, kidney diseases, and circulatory diseases. Mental health comorbidities included mood and anxiety, depressive, schizophrenia and delusional, and substance use disorders. References for more information on CDR and HSM can be found in notes for Table 1. Material/social deprivation is based on the Index of Material and Social Deprivation developed by the Institut national de santé publique du Québec (27).

In brief, the comparisons in Table 2 demonstrate that: (1) females were slightly more likely to get tested and be fully vaccinated, but slightly less likely to test positive; (2) older age was generally associated with a lower odds of ever testing and testing positive but a higher odds of being fully vaccinated; (3) greater material deprivation was associated with a higher odds of testing positive but a lower odds of ever testing and being fully vaccinated; and (4) worse health status (e.g., more co-morbidities and/or healthcare use) was generally associated with a higher odds of ever testing and being fully vaccinated but not testing positive (with some exceptions). There were also differences by regional health authorities.

### Syndromic surveillance

Syndromic surveillance may be useful as an early warning system for increases in COVID-19 transmission and/or as an additional way of monitoring COVID-19 transmission that is independent of testing behaviors/availability. It may also be useful for tracking other respiratory infections that are co-circulating with COVID-19.

**Figure 2:**
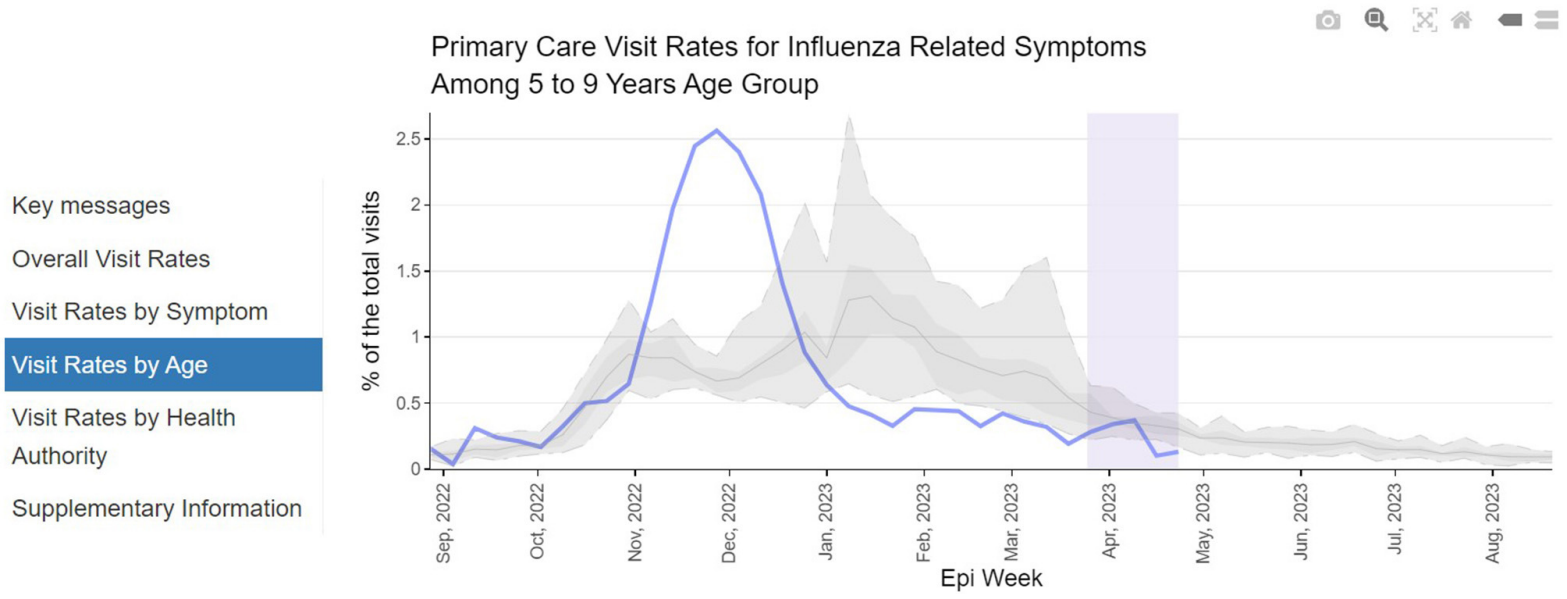
Respiratory disease syndromic surveillance dashboard. Screenshot presents proportion of primary care visits for influenza-related symptoms among individuals aged 5–9 years. Solid blue line represents current trend (2022/2023), while shaded gray lines/areas represent historical average and distribution from previous years (2010–2019).

We have used diagnostic codes in physician billing data to create an online dashboard for near “real-time” syndromic surveillance of primary care visits for COVID-19, acute respiratory infections (e.g., cough, cold), and influenza and pneumonia symptoms (6). The dashboard is available here: http://www.bccdc.ca/health-professionals/data-reports/respiratory-diseases. Trends are stratified by age and region and also compared to historical averages. The dashboard was implemented in preparation for the 2022/2023 winter season, during which syndromic surveillance demonstrated little COVID-19 circulation but large increases in other respiratory infections, particularly in younger age groups. Indeed, the rate of visits for clinically diagnosed influenza in younger individuals exceeded—and peaked earlier—than historical averages (Figure 2). Trends in these data were supported by other data sources (wastewater surveillance, laboratory testing), highlighting the validity of these data for use as syndromic surveillance.

### COVID-19 severity

**Figure 3:**
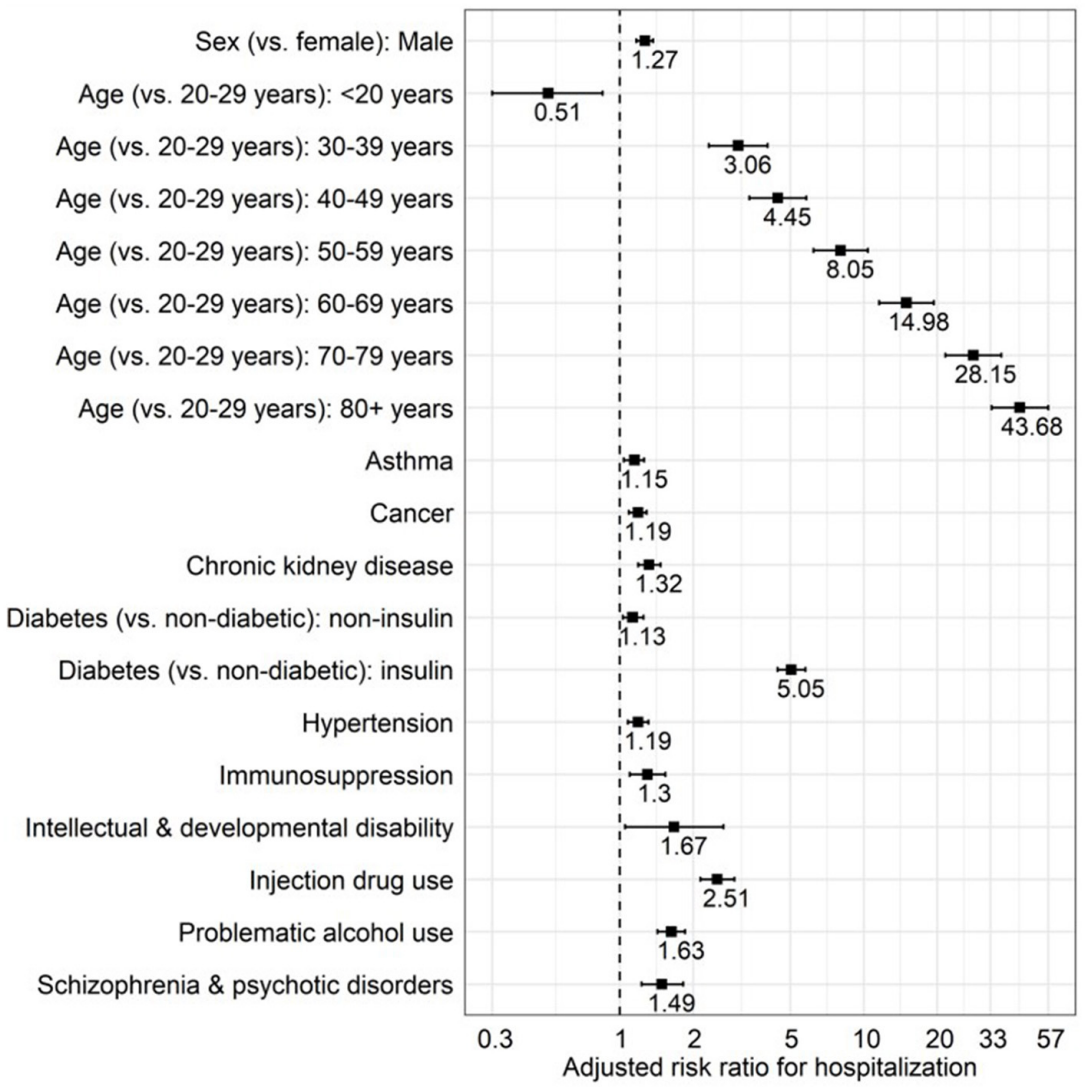
Characteristics associated with COVID-19 hospitalization among laboratory confirmed COVID-19 cases. Multivariable Poisson regression analysis with robust error variance. Analysis included all laboratory-diagnosed COVID-19 cases in British Columbia to 15 January 2021 (*N* = 56,874; 2,298 were hospitalized). Figure reproduced from “Mental Health and Substance Use Associated with Hospitalization among People with COVID-19: A Population-Based Cohort Study” by Velásquez García et al. (7) (CC BY 4.0); copyright 2021 by the authors; licensee MDPI, Basel, Switzerland.

Assessing characteristics associated with a higher risk of COVID-19 severe outcomes (e.g., hospitalization, ICU admission, death) is critical to identify higher-risk populations who can be prioritized for intervention, such as vaccination. A BCC19C analysis conducted early in the pandemic identified older age as the strongest risk factor for hospitalization—in addition to male sex and a range of co-morbidities (Figure 3) (7)—and these results were used to inform provincial vaccine rollout. Importantly, this analysis also identified mental health conditions and injection drug use as risk factors for hospitalization, highlighting the overlap between the two ongoing emergencies in BC (COVID-19 and unregulated drug poisoning). Later in the pandemic, this analysis was updated and the results highlighted the success of vaccination in mitigating the higher risk of severe outcomes associated with increasing age but also the need for additional protection (e.g., nirmatrelvir/ritonavir) in some populations (8).

Other BCC19C analyses have explored the relative clinical severity of SARS-CoV-2 variants of concern (VOCs). In an analysis conducted during Omicron and Delta co-circulation, we found Omicron to be associated with a 50% lower risk of hospitalization (aHR = 0.50; 95%CI = 0.43–0.59), a 73% lower risk of ICU admission (aHR = 0.27; 95%CI = 0.19–0.38), and a 5 day shorter hospital stay (ß = −5.03; 95% CI = −8.01−2.05) relative to Delta (9). Another study found differences in clinical severity between sub-variants of Omicron (BA.1, BA.2, BA.5) (10). Assessing all VOCs in the same analysis is challenging due to the short periods of co-circulation and potential for temporal biases, but our analysis attempting to account for these biases identified Delta as the most virulent VOC, followed by Gamma, Omicron, and Alpha (11).

We have also compared the population-level burden of COVID-19 hospitalizations to historical influenza seasons (12). This analysis found that rates of COVID-19 hospitalization per 100,000 adults were higher than influenza in the pre-Omicron period (low COVID-19 vaccine coverage, public health restrictions in place) but similar to influenza in the Omicron era (high COVID-19 vaccine coverage, relaxed public health measures). Among children (< 18 years of age), COVID-19 hospitalization rates were generally comparable or lower than influenza regardless of COVID-19 pandemic phase or vaccination status.

### Vaccine safety

Linked administrative data (emergency department visits, hospital discharges) can be used to monitor vaccine safety and identify adverse events of special interest (AESIs). BCC19C analyses have assessed safety signals in several ways. As part of a collaboration with the Global Vaccine Data Network (GVDN), we calculated background rates (i.e., prior to COVID-19 pandemic) for a range of AESIs and contributed to the creation of an online dashboard presenting these data (13). We have also published an individual-level analysis comparing the observed number of myocarditis events following COVID-19 vaccination to the number that would be expected based on these background rates. This analysis highlighted the overall safety of COVID-19 vaccines, but identified a higher than expected rate of myocarditis events following a second mRNA dose, particularly among young male mRNA-1273 recipients (14). These findings were supported by another BCC19C analysis identifying a higher odds of myocarditis for mRNA-1273 (vs. BNT162b2; aOR = 2.78; 95% CI: 1.67–4.62)—an association that was stronger among younger males but did not appear to be present among older individuals (>40 years of age) or females (15). These analyses supported the decision to preferentially recommend BNT162b2 for younger individuals. Promisingly, a more recent analysis suggested that myocarditis risk is lower with a booster dose, with no difference in myocarditis risk by vaccine type (16). In an unpublished analysis, we also found the rate of myocarditis following SARS-CoV-2 infection to be generally higher than after mRNA vaccination, particularly among females (regardless of age) and males older than 30—highlighting how vaccination can indirectly reduce risk of myocarditis.

### Vaccine effectiveness

We have analyzed the effectiveness of COVID-19 vaccines against infection and severe outcomes using the test-negative design. Early analyses conducted within a year of vaccine rollout confirmed that two doses of mRNA vaccines provided high effectiveness (>97%) against severe outcomes overall and across variants of concern (alpha, gamma, delta) (17). These early findings suggested that a longer interval between doses increased effectiveness and that effectiveness remained high >4 months following a second dose (17).

Additional vaccine effectiveness analyses are being conducted in immunocompromised populations who may experience lower vaccine effectiveness, such as people living with HIV (PLWH) or who inject drugs. In an analysis of PLWH, we found that vaccine effectiveness built up more slowly and waned more quickly compared to HIV-negative individuals, but that peak estimates were similar between the two populations (18).

### Unintended/societal consequences of the COVID-19 pandemic

Our analyses are exploring a range of potential societal/unintended consequences of the COVID-19 pandemic. These include negative impacts that go beyond the direct effect of viral infection on individual health. In an analysis exploring the impact on mental health, we found that trends in outpatient medical visits, emergency department visits and psychotropic drug dispensations increased following pandemic onset, particularly among adolescents (10–19 year olds) and females (19). Another study found the volume of ED visits reduce by 13% during the first 16-months of pandemic, with the biggest decrease among respiratory-related visits in the pediatric population (20).

Ongoing analyses are exploring other indirect negative impacts on health, such as those potentially arising from healthcare disruptions (e.g., delayed elective surgeries, reduced access to screening programs) or less health-seeking due to fear of exposure to COVID-19. Future reports will be posted at http://www.bccdc.ca/health-professionals/data-reports/societal-consequences-covid-19/health-care-services.

### Disparities in SES and SDOH

We are exploring SES/SDOH disparities in risk of COVID-19 infection and severe outcomes, as well as vaccine coverage. These analyses have found that COVID-19 cases tend to concentrate in areas with lower SES (e.g., lower income, education, housing suitability) and in areas with a higher proportion of people who are visible minorities, recent immigrants, and essential workers (21). In an analysis assessing the syndemic association between diabetes and SES/SDOH, COVID-19 diagnosis was found to be associated with geographic areas containing greater proportions of people of South Asian race/ethnicity, with lower educational status, and working in Trade/Transport occupations—and that these associations appeared to be stronger among individuals with diabetes (22). In another analysis examining the association between SES/SDOH and severe outcomes by VOC, the race/ethnicity distribution of severe COVID-19 outcomes was found to differ by VOC during co-circulation of Alpha and Gamma (11). Ongoing investigations are attempting to tease apart the mediating role of VOCs in the relationship between SDOH and severe outcomes.

### Post-acute outcomes following COVID-19, including long-COVID

Our team is using the BCC19C to characterize the long-term impact of COVID-19 on individual health and healthcare utilization. Initial work focused on the use of machine learning approaches to identify long-COVID patients in administrative datasets. In our first paper, an elastic net regression model leveraging several BCC19C datasets was used to create an algorithm for identifying long-COVID patients (23). Clinical data on individuals enrolled in post-COVID-recovery clinics in BC were integrated into the BCC19C to develop and validate the algorithm. The resulting algorithm was found to have high sensitivity (86%) and specificity (86%) for identifying long-COVID and has subsequently been used to generate a population-based cohort of ~25,000 patients. In the first analysis of this cohort, healthcare utilization among people with long-COVID was 2-times higher compared to people without during the first 6 months after acute infection, and remained elevated (although to a lesser degree) more than 1 year later (24).

Other analyses are assessing the long-term association between SARS-CoV-2 infection and specific non-respiratory sequelae. In one analysis, the risk of incident diabetes was higher among individuals who survived acute SARS-CoV-2 infection compared to uninfected controls (HR = 1.16, 95% CI: 1.06–1.28). This association was stronger among males and individuals with greater severity of acute SARS-CoV-2 infection (25).

## Discussion

In this paper, we have summarized the impact of the innovative BCCDL platform. This platform enabled real-time integration of surveillance, laboratory, health registries and healthcare administrative datasets during the COVID-19 pandemic. The BCC19C analyses of cohorts developed from this platform have facilitated deeper understanding of COVID-19 epidemiology and informed public health interventions and planning in BC. In addition, the platform has created new opportunities to monitor population health status and generate health intelligence in future emergencies and apply novel analytic techniques. Our initiatives complement similar efforts in other jurisdictions (4, 26), and together highlight their potential in jurisdictions without existing platforms. While implementation elsewhere may be limited by local availability of datasets, the overall concept of the platform could be considered broadly feasible given available resources, facilitating regulations, and successful stakeholder collaboration. Our efforts are continuing to evolve and act as an exemplar for similar platforms elsewhere.

Key strengths of the BCC19C are the comprehensive scope of available datasets, the ability to link individuals between de-identified datasets using a unique patient master key, and the dynamic/flexible nature of the BCCDL platform. Most datasets integrated into the platform—and used in BCC19C analyses—are population-based (i.e., cover all BC residents), thereby minimizing the potential for selection bias and maximizing generalizability of findings. In addition, the wide range of datasets permit measurement of most covariates and outcomes required for comprehensive COVID-19 analyses—with some datasets complementing each other to ensure more complete capture of specific measurements (e.g., multiple sources can be used to identify comorbidities or severe COVID-19 outcomes). The frequent refresh of available datasets allows for more timely analyses when needed and the flexibility of the BCCDL platform facilitates the addition of new datasets on an as-needed basis once appropriate approvals are obtained. Finally, the administrative datasets and provincial registries integrated in the BCCDL platform are not limited to COVID-19 and allow for selection of “control” individuals who have not accessed COVID-related services (e.g., people who have not tested or vaccinated for COVID-19). Ongoing and future investigations using this platform include assessing the association between COVID-19 and other long-term health outcomes (e.g., neurological and cardiovascular disorders), characterizing and monitoring long COVID, quantifying the impact of COVID-19 vaccination and VOCs on development of long-COVID, measuring the durability and severity of specific long-COVID symptoms (e.g., diabetes) and vaccine-associated myocarditis, assessing disruption of the pandemic on other health services (e.g., diabetes screening and diagnosis, HBV and HCV testing and treatment), exploring the reliability of machine learning prediction models trained on respiratory infection syndromic surveillance data, and use of mutation-level genomic SARS-CoV-2 data to develop polygenic risk scores for predicting severity of infection.

The BCC19C has some weaknesses, including limitations inherent to administrative/registry data, unavailable data elements, and possible errors in patient matching. Administrative data are collected for purposes other than public health surveillance and research, and secondary analysis of these data is limited by the elements available within each dataset and subject to errors in data collection/input. However, some of these limitations can be mitigated by use of multiple data sources to complement certain measures (e.g., laboratory test results to overcome limitations related to misclassification of administrative data). Examples of unavailable data elements that could improve and expand BCC19C analyses include individual-level information on race/ethnicity, immigration and other SES indicators (currently only available at the geographic level). Currently, the most recent geographic-level SES/SDOH data is from the 2016 census but will be updated once 2021 census data becomes available. Measurement of health conditions in administrative data is based on healthcare visits and misses individuals who have not sought care. Further, some characteristics cannot be identified in administrative data with high sensitivity and specificity, such as smoking and certain anthropomorphic measures (e.g., Body Mass Index). Future linkage to survey data may improve capture of certain variables, including SES and specific comorbidities, in a subset of individuals. The patient matching algorithm requires review, validation, and recalibration after introduction of new datasets—raising the potential for errors in between validation cycles. Finally, some datasets are more lagged than others, either due to more infrequent refresh schedules (e.g., some datasets are only updated monthly/annually) and/or inherent lag in the source data (e.g., some datasets are refreshed frequently but with data that is lagged by a month or more), limiting the ability to conduct more timely analyses. However, many BCC19C analyses are more retrospective in nature and do not require “real-time” data availability. Within this context, there is a need for assessment and validation of real-time surveillance measures using data which are more timely, such as laboratory information, prescriptions and medical visits.

## Ethics statement

The studies involving humans were approved by Research Ethics Board of the University of BC (Approval # H20-02097). The studies were conducted in accordance with the local legislation and institutional requirements. Written informed consent for participation was not required from the participants or the participants' legal guardians/next of kin because this study was performed using de-identified data routinely collected as part of public health surveillance and/or routine healthcare encounters. Patient consent was not required in accordance with the Canadian Tri-Council Policy Statement: Ethical Conduct for Research Involving Humans article 5.5B.

## Author contributions

SM, JA, NJ, and MN led and/or contributed to the establishment of the BC COVID-19 Data Library (BCCDL) platform, including dataset acquisition, linkages, and maintenance. NJ led the development of the BC COVID-19 Cohort (BCC19C) including ethics approval, funding, and other support. MCo led the governance aspects of the BCCDL and manages the PHSA COVID-19 Analytic Network. MCh, JW, and AB were involved in supporting BCC19C access and analyses for BCC19C users, including development of technical documents, data dictionaries, and administrative processes. MCh, ZN, KS, HV, SH, DR, BA, MT, MZ, JL, CR, HS, NJ, YA, SS, MB, and JW contributed to the published papers and dashboards described in the manuscript. JW conducted the analysis in Table 2. HS, MT, and AF are involved in the curation/maintenance of specific datasets integrated in the BCCDL. JW wrote the first draft of the manuscript with support from SH. All authors reviewed and provided critical feedback on the manuscript. All authors contributed to the article and approved the submitted version.

## BCC19C collaborators

James Wilton, Jalud Abdulmenan, Mei Chong, Ana Becerra, Mehazabeen Najmul Hussain, Sean P. Harrigan, Héctor Alexander Velásquez García, Zaeema Naveed, Hind Sbihi, Kate Smolina, Marsha Taylor, Binay Adhikari, Moe Zandy, Solmaz Setayeshgar, Julia Li, Younathan Abdia, Mawuena Binka, Drona Rasali, Caren Rose, Michael Coss, Alexandra Flatt, Seyed Ali Mussavi Rizi, Naveed Zafar Janjua, Mike Irvine, Braeden Klaver, Prince Adu, Hasina Samji, Georgine Cua, Chad Fibke, Bushra Mahmood, Stanley Wong, Angela Yao, Geoffrey McKee, Monika Naus.
