# Supplementary material for: Cohort profile: the British Columbia COVID-19 Cohort (BCC19C)—a dynamic, linked population-based cohort
Source: Front Public Health. 2024 Feb 21;12:1248905. doi: 10.3389/fpubh.2024.1248905 (PMC10914982; doi:10.3389/fpubh.2024.1248905)
Supplement: Supplementary file 1 [file Data_Sheet_1.docx]

Supplementary Material

Cohort profile: The British Columbia COVID-19 Cohort (BCC19C) – a dynamic, linked population-based platform

**Supplemental Table 1. Data Sources integrated within the BC COVID-19 Cohort (BCC19C)**

| **British Columbia Centre for Disease Control (BCCDC), Provincial Heath Services Authority (PHSA) and Regional Health Authority data sources:** | **Data Date Ranges:** |
| --- | --- |
| Integrated COVID-19 laboratory dataset (SARS-CoV2 tests from private/public labs) ^S1^ | Jan,2020-onward |
| COVID-19 surveillance case data (information collected on all probable/confirmed cases as part of public health follow up) ^S2^ | Jan,2020-onward |
| Provincial COVID-19 Monitoring Solution (critical and non-critical care hospital census data) ^S3^ | Jan,2020-onward |
| Provincial Immunizations Registry (COVID-19 vaccination data) ^S4^ | Dec,2020-onward |
| Provincial Laboratory Information Solution (laboratory tests from private/public labs) ^S5^ | Jan,2020-onward |
| Public Health Reporting Data warehouse (Influenza laboratory tests) ^S6^ | Jan,2008-onward |
| Emergency department visits (hospital-based and community-based ambulatory care) | Mar,2020-onward |
| **Ministry of Health (MoH) Administrative Data Sources:** | **Data Date Ranges:** |
| Client Roster (CR) (registry of enrollment in the universal public health insurance plan including residential history) ^S7^ | 2008/9-onward |
| Discharge Abstracts Database (DAD) (hospital discharge records) ^S8^ | 2008/9-onward |
| Medical Services Plan (MSP) (physician diagnostic and billing data for services provided through universal public health insurance plan) ^S9^ | 2008/9-onward |
| PharmaNet (Pharma) (prescription drugs dispensed from community pharmacies, includes medications covered by public and private insurance plans) ^S10^ | 2008/9-onward |
| BC Vital Statistics (VS) (deaths registry)^S11^ | 2008/9-onward |
| National Ambulatory Care Reporting System (NACRS) (hospital-based and community-based ambulatory care) ^S12^ | 2011/12–onward |
| Chronic Disease Registry ^S13^ | 2008/9-2018/19 |
| 811 Calls (respiratory calls only) ^S14^ | 2014-onward |
| Health System Matrix ^S15^ | 2018/19-onward |
| Population Grouper Methodology ^S16^ | 2008/9-onward |

**Supplementary References**:

1. British Columbia Centre for Disease Control [creator]. Integrated COVID-19 laboratory dataset (SARS-CoV2 tests from private/public labs). Public Health Reporting Data Warehouse, British Columbia Centre for Disease Control [publisher] (2020). 2021.
2. British Columbia Centre for Disease Control [creator]. COVID-19 surveillance case data. British Columbia Centre for Disease Control [publisher]. (2020). 2021.
3. Provincial Health Services Authority [creator]. Provincial COVID-19 Monitoring Solution. Provincial Health Services Authority [publisher]. (2020). 2021.
4. Provincial Health Services Authority [creator]. COVID-19 vaccination data. Provincial Immunizations Registry, Provincial Public Health Information Systems [publisher]. (2020). 2021.
5. Provincial Health Services Authority [creator]. Provincial Public Health Information Systems [publisher]. (2020). 2021.
6. British Columbia Centre for Disease Control [creator]. Respiratory datamart, Public Health Reporting Data Warehouse, British Columbia Centre for Disease Control [publisher] (2020). 2021.
7. British Columbia Ministry of Health [creator]. Client Roster (Client Registry System/Enterprise Master Patient Index). British Columbia Ministry of Health [publisher]. Data Extract. MOH (2020). 2021. <https://www2.gov.bc.ca/gov/content/health/health-forms/online-services>
8. British Columbia Ministry of Health [creator]. Discharge Abstract Database (Hospital Separations). British Columbia Ministry of Health [publisher]. Data Extract. MOH (2020). 2021. <https://www2.gov.bc.ca/gov/content/health/health-forms/online-services>
9. British Columbia Ministry of Health [creator]. Medical Services Plan (MSP) Payment Information File. British Columbia Ministry of Health [publisher]. Data Extract. MOH (2020). 2021.<https://www2.gov.bc.ca/gov/content/health/health-forms/online-services>
10. British Columbia Ministry of Health [creator]. PharmaNet. British Columbia Ministry of Health [publisher]. Data Extract. MOH (2020). 2021. <https://www2.gov.bc.ca/gov/content/health/health-forms/online-services>
11. BC Vital Statistics Agency [creator]. Vital Statistics Deaths. BC Vital Statistics Agency [publisher]. Data Extract. BC Vital Statistics Agency (2020). 2021. <https://www2.gov.bc.ca/gov/content/health/health-forms/online-services>
12. British Columbia Ministry of Health [creator]. National Ambulatory Care Reporting System. British Columbia Ministry of Health [publisher]. Data Extract. MOH (2020). 2021.

<https://www2.gov.bc.ca/gov/content/health/health-forms/online-services>

1. British Columbia Ministry of Health [creator]. Chronic Disease Registry. British Columbia Ministry of Health [publisher]. Data Extract. MOH (2020). 2020.

<https://www2.gov.bc.ca/gov/content/health/health-forms/online-services>

1. British Columbia Ministry of Health [creator]. 811 calls. British Columbia Ministry of Health [publisher]. Data Extract. MOH (2020). 2021.

<https://www2.gov.bc.ca/gov/content/health/health-forms/online-services>

1. British Columbia Ministry of Health [creator]. Health System Matrix. British Columbia Ministry of Health [publisher]. Data Extract. MOH (2020). 2021.

<https://www2.gov.bc.ca/gov/content/health/health-forms/online-services>

1. British Columbia Ministry of Health [creator]. Population Grouper Methodology. British Columbia Ministry of Health [publisher]. Data Extract. MOH (2020). 2021.

<https://www2.gov.bc.ca/gov/content/health/health-forms/online-services>
